# Athletes are better at peripheral colour detection

**DOI:** 10.1177/03010066251414084

**Published:** 2026-02-13

**Authors:** Sidney Uden-Taylor, Anna Metzger, Matteo Toscani

**Affiliations:** 1 6657 School of Psychology, Bournemouth University, Poole, UK

**Keywords:** colour, perceptual learning, spatial vision, crowding/eccentricity

## Abstract

This study examined how background context and athletic experience influence peripheral colour detection. Twenty-six participants – outdoor athletes, indoor athletes and non-athletes – completed a colour detection task with human figure or circle stimuli on sport-specific indoor and outdoor scenes. Outdoor athletes showed superior detection across conditions. Crucially, this advantage was not restricted to sport-specific stimuli or backgrounds, suggesting that perceptual learning in athletes generalises across contexts. This quasi-experimental study compared naturally occurring sport groups rather than assigning participants to conditions, allowing investigation of experience-related differences in peripheral processing. The findings provide evidence that perceptual expertise in sport reflects more than reaction speed, highlighting improvements in low-level perception. They also demonstrate that visual plasticity persists beyond critical developmental periods: repeated exposure to complex, unpredictable outdoor environments appears sufficient to shape perceptual abilities later in life.

## Introduction

The architecture of the visual system varies systematically with retinal eccentricity (Strasburger et al., 2011). Cone photoreceptor density is maximal in the foveal region and declines rapidly with increasing distance from the centre of gaze, giving way to a rod-dominated periphery. This non-uniform architecture contributes to differences in perceptual fidelity across the visual field causing differences in visual acuity, contrast sensitivity and colour sensitivity (Hansen et al., 2009; Rovamo et al., 1978; Weale, 1953; Weymouth, 1958) and in the appearance of basic visual features such as spatial frequency, luminance or chromatic saturation or numerosity (Davis, 1990; Greenstein & Hood, 1981; McKeefry et al., 2007; Valsecchi et al., 2013).

Peripheral vision within sport is viewed as a vital tool in perceiving the surroundings to enable a successful performance (Schumacher et al., 2019). Despite the high resolution of foveal vision, athletes need to use peripheral vision to provide them with a broader environmental awareness. Athletes within team-based sports use periphery to detect movements of opponents and opposition (Klostermann et al., 2020; Vater et al., 2020) and aid in the anticipation of player movements (Williams et al., 2002). Extensive use of peripheral vision in athletes may enhance peripheral processing abilities through perceptual learning.

Perceptual learning refers to the stable change in the perception of a set of a certain stimuli following practice or experience with this stimuli (Gibson, 1969). Given the importance of peripheral vision for sport, athletes may develop more effective and perceptive abilities to detect and respond to peripheral stimuli. Supporting this, Stine et al. (1982) reported that athletes exhibit superior visual abilities compared to non-athletes, including peripheral acuity and extent of the visual field, and importantly, that these abilities are trainable through consistent practice and experience, highlighting the role of perceptual learning in athletic contexts. Furthermore, athletes have higher peripheral awareness – the ability to respond to relevant peripheral information, and faster reaction times than non-athletes (Christenson & Winkelstein, 1988; Mathe et al., 2023).

However, these studies rely on test batteries that do not allow for isolating visual performance. For instance, peripheral awareness was measured by the number of peripheral targets a participant can hit within 60 s (Mathe et al., 2023). This measure depends not only on the ability to detect stimuli but also on the ability to respond rapidly and sequentially. Similarly, as shown in by a recent systematic review (Vater & Strasburger, 2021), studies reporting peripheral vision tests in athletes typically do not involve eye-tracking to control for the use of peripheral vision. Such tests focus on performance measures to test peripheral monitoring/awareness, peripheral reaction times or extent of the visual field rather than perception. Another recent review of over 4000 studies (Lochhead et al., 2024) showed that while more than 40 of these studies included tests of peripheral vision, such studies (e.g., Ward & Williams, 2003) typically measure peripheral awareness rather than perception in peripheral vision.

Although research focussed on peripheral awareness, there are a few studies comparing peripheral perception between athletes and non-athletes using perceptual measures such as detection, as reported by a review of 22 studies (Ong, 2015). However, they generally did not find differences. For instance, one study examining detection and reaction times reported no differences in detection performance, although athletes showed faster reaction times (Zwierko, 2007). Similarly, Nascimento et al. (2021) showed that futsal players outperformed non-athletes only in a task measuring predictive visual-motor coordination and found no differences more purely perceptual tasks such as reaching for a peripheral target after it changed to a certain colour. These findings align with broader evidence summarised by the Ong review (2015).

Notably, these studies examined indoor sports and did not assess colour perception, which decays with eccentricity more than luminance perception (e.g., Hansen et al., 2009).

The present study takes a novel approach through examining colour perception within the periphery of different types of athletes and non-athletes. Colour perception systematically declines with eccentricity and may be particularly subject to compensatory mechanisms – such as perceptual learning – in populations who rely on it, like athletes. In fact, while cone density decreases with eccentricity leading to lower detection performance (e.g., Hansen et al., 2009), colour detection within game-based sport is vital in identifying the opposition, ball and teammates (Burnell & Thompson, 2021; Olde Rikkert et al., 2015; Schleifer & Tamir, 2023). However, research supporting the role of colour perception on sport performance is not conclusive as several studies found no performance difference associated with the colour of players uniform (for a review see Goldschmied et al., 2023).

In this study, we investigate colour detection in peripheral vision among athletes and non-athletes, using different colour backgrounds and target stimuli that are either human-like (typical experience in sport settings) or circular. Importantly, the stimuli and backgrounds are specific to different sports, allowing us to examine how much perceptual learning is tailored to each sport. The study includes participants from both l and futsal due to these sports requiring high levels of tactical complexity which needs the athletes to observe their surroundings very frequently due to limited space and proximity to other players. These sports also have highly unstable environments, meaning these athletes must adapt to situations that can appear anywhere across the visual field. We recruited outdoor and indoor athletes because outdoor sports such as l, soccer and rugby are played on much larger fields compared to indoor sports. This requires athletes to monitor a wider area, including teammates, opponents and the ball, which is where peripheral vision becomes crucial. If improved colour detection is specific to peripheral vision and results from experience (perceptual learning), we expect to find it more pronounced in outdoor athletes.

While our approach allows us to investigate experience-related differences in peripheral processing, it does not allow us to draw inferences about causal direction, since participants were not randomly assigned to experimental groups.

## Method

### Participants

Twenty-six participants (3 females) completed the study (9 in the indoor group, 8 in the outdoor group and 9 non-athletes), aged 20–22 (*M* = 21.1, *SD* = 0.40). To qualify as an athlete each participant from both the non-athlete and athlete group had to have participated in their chosen sport for over a period of 3 years. Due to considerable inconsistency in how elite/expert athletes are defined in sport psychology research, a conventional classification of training levels is needed for comparison purposes (Swann et al., 2015). According to McKay's (McKay et al., 2021) framework the outdoor and indoor athletes were gathered through a Tier 2 level, where all athletes trained very regularly and most competed at a university level. The non-athletes would fit into Tier 0, where there is incidental physical activity at most. Age distributions were similar across groups, with indoor athletes (*M* = 21.22, *SD* = 0.44), outdoor athletes (*M* = 21.11, *SD* = 0.33) and non-athletes (*M* = 21.56, *SD* = 0.53). Weekly training hours were also similar across groups. Indoor athletes reported training an average of 4.56 h per week (*SD* = 1.13), while outdoor athletes trained an average of 4.44 h per week (*SD* = 0.74). To avoid the cross contamination of the type of sports the athletes perform, we made sure they only competed in their specific sport faction (indoor or outdoor). Because of the similar age and training across athlete groups, differences in perceptual performance are likely attributable to the type of sport rather than to age or training.

All reported normal or corrected-to-normal vision and no colour blindness. Participants with any known ocular diseases or neurological conditions were excluded from the study. We did not explicitly assess vision, colour blindness or neurological conditions, relying solely on self-reports.

The study was approved by the ethics committee of Bournemouth University and conducted in accordance with the 2013 Declaration of Helsinki, except for preregistration.

### Apparatus

#### Stimuli

**Figure 1.**
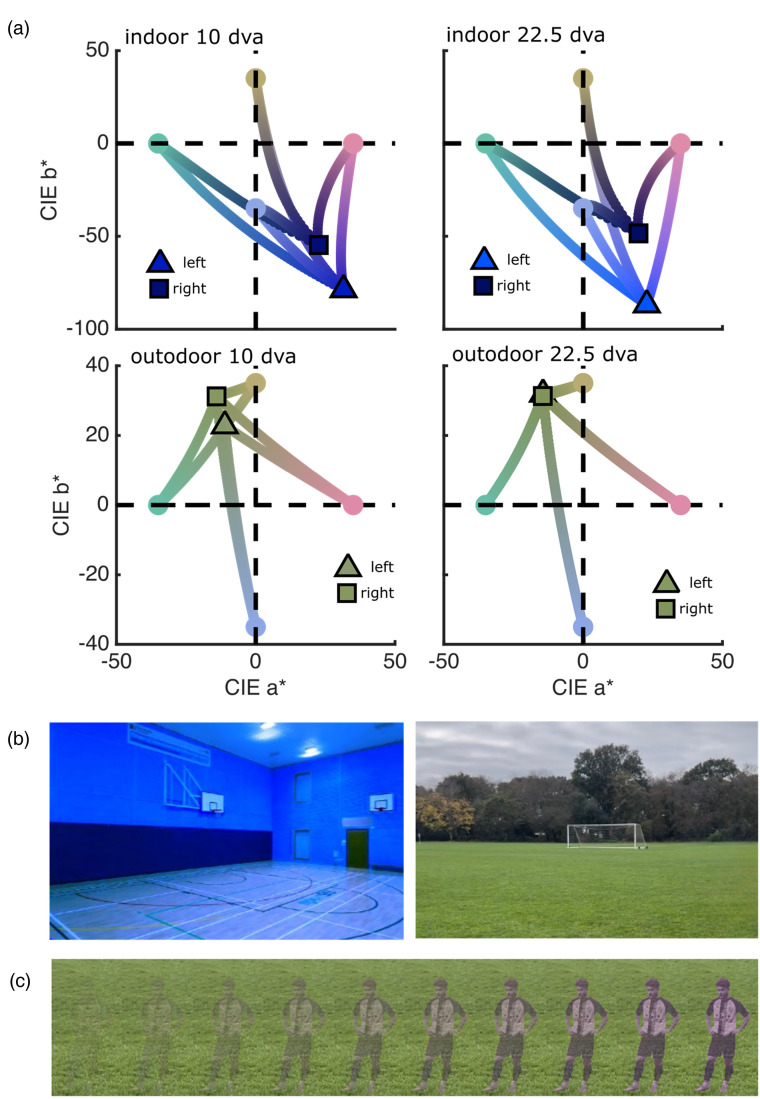
Stimuli. (a) Stimuli in CIE L*a*b* colour space. Circles represent the four selected colours; the triangle and square represent the average background colour where the human figure was placed, on the left and right sides of the background, respectively. The two diagrams on the left show colours for stimuli presented at 10 degrees of visual angle (dva) eccentricity, while the diagram on the right shows colours for 22.5 dva. The background colours and resulting weighted averages differ because the area of the background on which the stimulus appears varies with eccentricity. Top diagrams are for the indoor scene bottom for the outdoor. The lines connecting each circle to the corresponding background colour indicate how the stimulus colour gradually shifts towards the background colour as w decreases. Each data point in colour space is rendered using its corresponding rgb colour. (b) Indoor and outdoor background. (c) Example of the human figure changing visibility with w increasing from left to right in linear steps, from 0.05 to 0.4, for initial colour set to a* = 35, b* = 0, placed on the outdoor background.

We used a coloured circle and human figure. The human figure was a photograph of the first author as a sports’ player (Figure 1(c)), presented so that their height was around 3 degrees of visual angle (dva). The circle was rendered to have the same number of pixels as the human figure, to promote similar visibility and the same average colour.

Two different backgrounds were used representing both an indoor background and an outdoor background (Figure 1(b))*.* The outdoor background was selected based upon guidance from the l Association, as their research indicates that over 87% of l pitches within the UK are grass and nature filled (The Football Foundation, 2021). The Indoor background was chosen through guidance from Sport England who suggest that indoor venues in England are coated in blue (86 BG 43/321) paint (Sport England, 2012).

**Figure 2.**
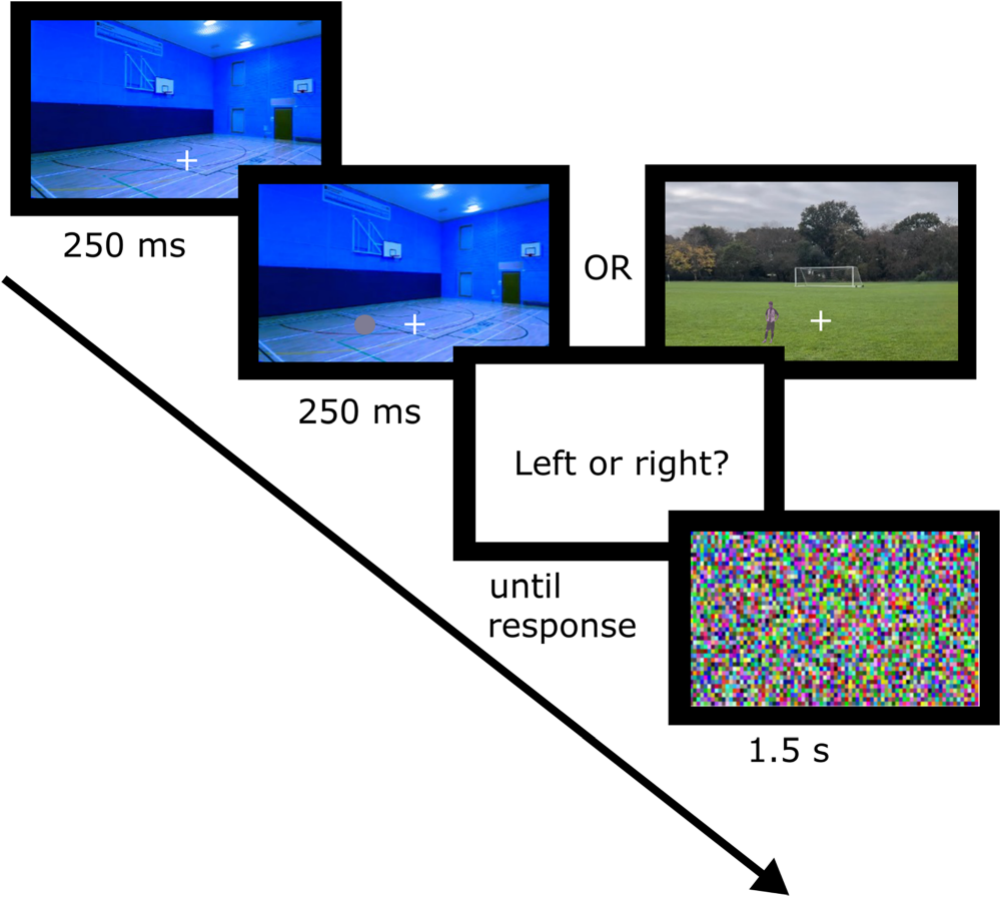
Procedure.

The CIE L*a*b* colour space was used to define four colours along cardinal directions of the a* and b* axes, 35 units away from the centre (circles in Figure 2(a)). The human figure transformed from rgb to L*a*b* coordinates, then all values of the a* and b* channels were set to the a* and b* coordinates of each of the four colours, the L* component was left untouched (average L* = 30), then converted back to rgb. The colour of the circle was set to the average rgb of the human figure. To reduce visibility, the stimuli were blended with the background using weighted averaging, where the weight *w* was proportional to visibility. Specifically, when *w* = 1, the stimulus completely replaced the background, and when *w* = 0, only the background was visible (an example is shown in Figure 1(c)). Averaging was done in RGB, which resulted in a curved line in CIE L*a*b* space. Because the background colour differed between indoor and outdoor conditions – and also slightly between the left and right sides (as the stimulus could be randomly placed on either side) – as well as between lower and higher eccentricities, decreasing www defined different trajectories in colour space (Figure 2(a)).

#### Colour Calibration

We used a 24″ HP LA2205 W6 monitor (1920 × 1080 pixels, 60 Hz refresh rate) to present the stimuli. We performed a standard calibration procedure (Gil Rodríguez et al., 2022; Milojevic et al., 2018; Toscani et al., 2019) to linearise the screen and ensure accurate colour display. We measured the gamma curves of each channel and their chromaticity with the Spyder 4 colorimeter (Datacolor, Lawrenceville, NJ). This colorimeter performs on par with professional, high-cost photometers (ColorCal MKII and PR-670) in measurement accuracy (Lin et al., n.d.). Our screen had the following chromaticity: red primary CIE xyY coordinate (x: 0.658, y: 0.341, Y: 17.95 cd/m^2^), green primary CIE xyY coordinate (x: 0.316, y: 0.653, Y: 41.86 cd/m^2^) and blue primary CIE xyY coordinate (x: 0.145, y: 0.089, Y: 8.04 cd/m^2^). The gamma exponents were 2.15, 2.2 and 2.21, for the red, green and blue channel, respectively.

#### Procedure

Participants were seated at a fixed distance of 40 cm from the display to ensure accurate retinal eccentricities of 10° and 22.5°. The experiment was conducted in a soundproof dark room to eliminate ambient light and maintain fixation on the central point. Each stimulus was presented for 250 ms at either 10° or 22.5° eccentricity on the left or right side of fixation, in semi-randomised order – to ensure participants could not directly look at it (Hansen et al., 2009). Each condition (human figure vs circle, indoor vs outdoor, 10 vs 22.5 dva, four colour directions) was repeated 30 times per side, for a total of 2 × 2 × 4 × 2 × 30 × 2 = 1920 trials. Participants were required to maintain fixation on the cross in the centre and indicate the stimulus location using the left and right arrow keys on a keyboard (2AFC task). To counteract aftereffects, a dynamic mask – comprising randomly generated RGB pixel values that changed every frame at the screen's 60 Hz refresh rate – was presented for 1.5 s after the participant's response in each trial (Figure 2). The experiment lasted approximately 2 hours, and participants were allowed to take breaks whenever they wished.

Stimulus contrast was adaptively changed with using a Bayesian adaptive staircase (Watson & Pelli, 1983), to accurately estimate detection thresholds. Depending on participants’ responses, the stimulus colour and its contrast with the background varied along the trajectories shown in Figure 1(a). Stimuli were presented on either the left or right side of the scene, with each side used half of the time, in a semi-random order. We ran one staircase per condition, also in semi-random order. After 30 trials per side, the staircase was terminated. Based on the 60 trials (30 per side) per condition, thresholds were estimated with the psignifit MATLAB toolbox (Schütt et al., 2016). For our adaptive staircase, we specified a cumulative Gaussian psychometric function, with the starting threshold set at 0.75 based on the 2AFC task, the slope fixed at 0.1 as determined from a pilot, and the lapse and guess rates set to stabilise the fits, with the lower asymptote fixed at 0.5 (chance level) and the upper asymptote at 1. We used Pearson's chi-square test to assess goodness of fit. Data were divided into 11 bins (5 bins with 6 observations, 6 with 5), yielding 8 degrees of freedom and a critical value of χ^2^ = 15.5. Observed χ^2^ values ranged from 0 to 5.46 (*M* = 1.77), indicating that in no condition did the psychometric function predictions significantly deviate from the observed data.

## Results

We conducted a mixed analysis of variance (ANOVA) with participants’ group as between participants factor, and colour, eccentricity, background-type and stimulus type as within factors. To control for familywise error due to the multiple tests involved in the ANOVA (Cramer et al., 2016), especially when many factors are tested, we correct *p*-values following the Bonferroni procedure as suggested by Cramer et al. (2016). Results are reported with adjusted *p*-values.

**Figure 3.**
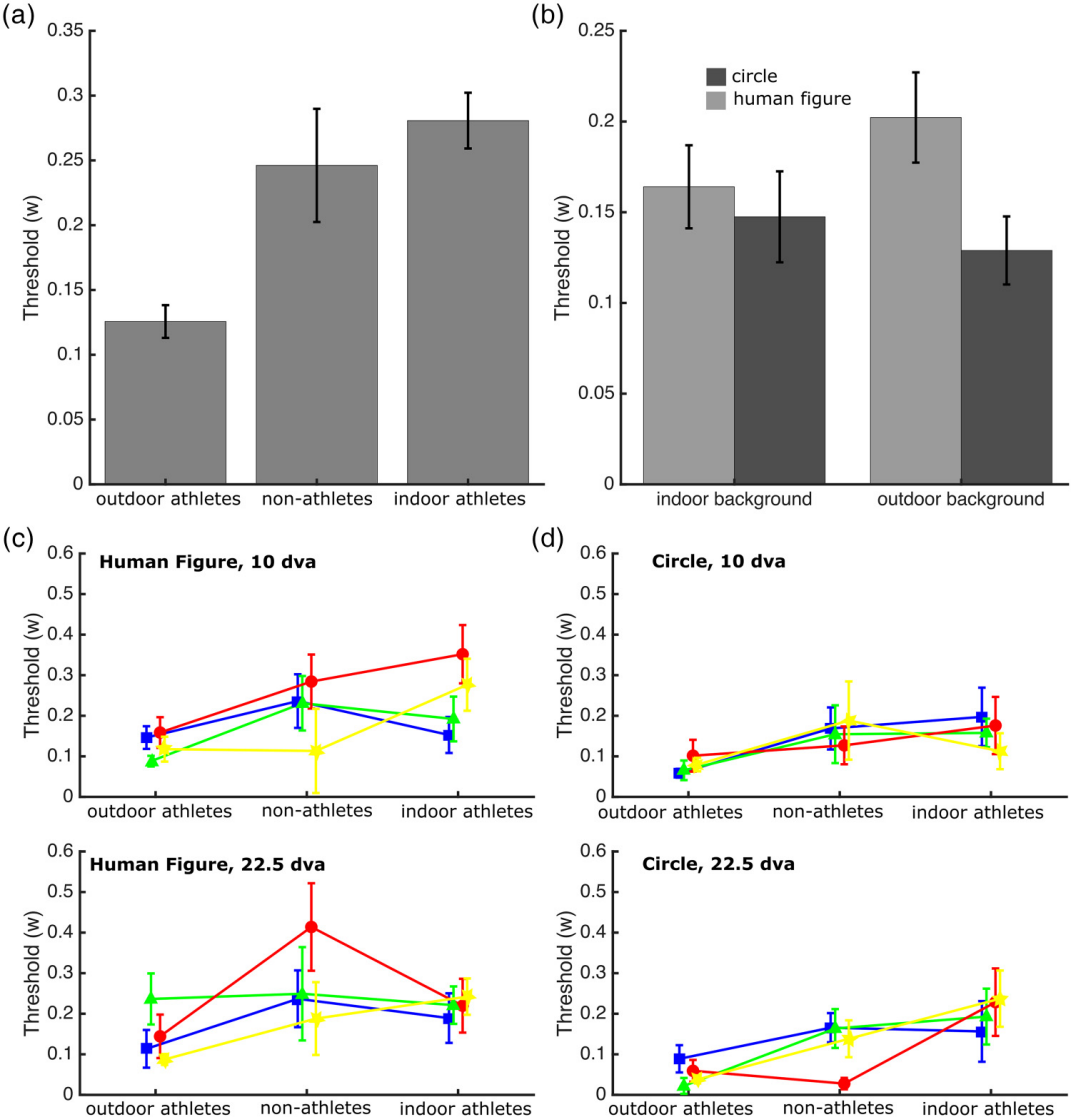
(a) Threshold (y-axis) as a function of group effect (y-axis). (b) Threshold (y-axis) as a function of type of background (x-axis) and shape of stimulus (different colours). (c) Threshold (y-axis) as a function group (x-axis), colour (different coloured symbols and lines indicating the different colours, red circles, green triangles, blue squares and yellow stars), stimulus type (human figure for the left diagrams, circle for the right diagrams) and background type (top diagrams for 10dva, bottom for 22.5 dva). The error bars represent the standard error of the mean.

Analysis of variance revealed a main effect of group (*F*_(2,23)_ = 3.82, *p* = .037, η_*p*_^2^ = .249). As shown in Figure 3(a), outdoor athletes exhibit lower thresholds for colour discrimination, indicating greater ability to detect colours in peripheral vision. The ANOVA also revealed a significant interaction between the type of stimulus and type of background (*F*_(1,23)_ = 8.420, *p* = .024, η_*p*_^2^ = .268). As shown in Figure 3(b), while the human figure is as easily discriminable as the circle on the indoor background, it becomes significantly more difficult to distinguish on the outdoor background.

Analysis of variance also revealed a significant four-way interaction between colour, eccentricity, group and stimulus type (*F*_(6,69)_ = 3.09, *p* = .02, η_*p*_^2^ = *.*21). Figure 3(c) shows that, at higher eccentricity (bottom diagrams), non-athletes exhibit poorer discrimination performance for the red colour when the stimulus is a human figure (left diagram) compared to a circle (right diagram). This suggests that at higher eccentricity non-athletes struggle more than athletes to spot the human figure while athletes’ experience with it may help them compensate. However, this result is complex and hard to interpret, and the differences between colours are particularly unclear. All other main effects and interactions were not significant. Notably, the interactions between group and stimulus type (*F*_(2,23)_ = 0.675, *p* = .518, *η_*p*_^2^* = .056), group and background type (*F*_(2,23)_ = 0.248, *p* = .783, *η_*p*_*^2^ = .020), and their three-way interaction (*F*_(2,23)_ = 0.715, *p* = .500, *η_*p*_^2^* = .059) were also not significant and had a small contribution to the total variance, as indicated by the small effect sizes. In fact, an a priori power analysis shows that, given our sample size and design, we would only be able to detect interactions with effect sizes of at least η_*p*_^2^ = .19. To detect a significant interaction with effect sizes comparable to those observed in the null results discussed above, a much larger sample would be required; for example, with η_*p*_^2^ = .020, at least 252 participants would be needed. Although these are null results, they suggest that the learned advantage of outdoor athletes generalises across different stimuli and environments; otherwise, we would observe an interaction with background type, with outdoor athletes outperforming the other groups predominantly in the outdoor background condition.

## Discussion

We found that outdoor athletes performed better in detecting peripheral stimuli, outperforming both indoor athletes and non-athletes. However, the findings show that performance was not better in sport-specific backgrounds or with sport-specific stimuli, suggesting that perceptual learning may be more generalisable, rather than context-dependent. We speculate this because outdoor athletes outperformed the other groups even when the stimuli were presented on an indoor background, suggesting that their improvement in peripheral detection performance generalises to environments different from those in which they trained their peripheral vision.

These results provide evidence for perceptual learning for outdoor athletes when compared to both indoor athletes and non-athletes. This extends the findings of Mathe et al. (2023), who used wire test batteries and identified signs of perceptual learning through combined measures of reaction time and detection accuracy, though these measures may have been confounding, as faster responses could have influenced perceived improvements in perceptual performance. In contrast, our study focused exclusively on a low-level perceptual task such as colour detection, offering specific evidence in support of perceptual learning.

It is well established that the brain exhibits high plasticity, adapting and being shaped significantly through early-life experiences (Cisneros-Franco et al., 2020). This suggests that visual perception is not fully innate but rather learned through exposure during critical developmental periods (Hirsch & Spinelli, 1971). While this has been well-documented in early formation stages, there is comparatively limited evidence supporting such plasticity beyond these periods. However, our findings indicate that the brain remains adaptable even outside of early development, highlighting the potential for continued perceptual learning later in life.

Previous research has often failed to demonstrate perceptual advantages for athletes in peripheral detection tasks, which contrasts with our finding that outdoor athletes outperformed non-athletes. For example, Zwierko (2007) and Zwierko et al. (2008) tested handball players, both in indoor contexts, and reported no differences in peripheral detection between athletes and controls. Similarly, Nascimento et al. (2021) found that futsal players outperformed non-athletes only in a predictive visuomotor coordination task, but not in more purely perceptual tasks such as peripheral detection. However, Zwierko et al. (2010) reported shorter visual evoked potential latencies and faster visual processing in volleyball players. While volleyball is played indoors on a relatively small field, the authors argue that the differences they found in visual processing between volleyball athletes and non-athletes may reflect the particularly dynamic and visually demanding nature of the sport. By contrast, our study revealed clear advantages for outdoor athletes, whose sports require continuous monitoring of wider visual environments. Taken together, these comparisons suggest that the demands of outdoor versus indoor sports are key factors in determining whether athlete advantages in peripheral vision emerge.

Superior performance of outdoor athletes is consistent with to findings by Norman et al. (2005, 2015) who reported that outdoor athletes show greater accuracy in perceiving distances and exhibit heightened spatial perception, likely as a result of continuous exposure to complex and variable natural environments. These studies support the interpretation that the dynamic, unpredictable nature of outdoor settings provides richer perceptual input, in particular a broader visual field. As such, our findings highlight the critical role of environmental context in shaping perceptual learning and suggest that outdoor sports may offer optimal conditions for enhancing peripheral visual processing.

However, our findings suggest that athletes’ visual performance did not differ when responding to stimuli presented in their own sport-specific context compared to that of another sport. Instead, it appeared that the enhanced perceptual abilities observed in outdoor athletes generalise across all background environments. This may suggest that once perceptual learning has occurred, it broadens visual processing capabilities beyond familiar or sport-specific settings. These results align with research by Xiao et al. (2008) and Faubert (2013), both of whom demonstrated that perceptual learning and cognitive advantages in athletes can transfer across tasks and contexts, supporting the idea of a flexible, domain-general visual learning mechanism. A potential confound, though, is cross-sport participation. While we made sure they only competed in their specific sport faction (indoor or outdoor), we did not control for non-competitive play. For instance, l players may often engage in futsal during off-season periods because of the ease of organising games and accessing facilities, whereas the reverse (futsal players regularly playing outdoor l) is less common. This asymmetry could create partial overlap between the groups and may account for why we observed a more generalised advantage across backgrounds rather than an exclusively outdoor-specific effect.

We observed no significant interaction between group and eccentricity, indicating that colour detection did not vary across peripheral positions in this study. This null effect may be due to the limited range of eccentricities tested, which likely did not extend far enough into the peripheral visual field to elicit measurable differences in perceptual learning between athlete and non-athlete groups (Poltavski & Biberdorf, 2014). Previous studies have consistently shown that perceptual performance (including colour detection, pattern recognition and detail perception) declines with increasing eccentricity, a trend not shown within the current results (Strasburger et al., 2011). To more accurately assess the influence of perceptual learning on peripheral colour detection, future research should consider using a broader visual field and presenting stimuli at greater eccentricities from the fovea.

Although our results did not show a significant main effect supporting improved performance of athletes on their sport-specific backgrounds, a significant interaction emerged between stimulus shape and background. Specifically, performance on the Human Figure stimulus in the outdoor background was particularly poor on the outdoor background. This may be due to stronger crowding caused by both the complexity of the outdoor scene and the internal structure caused by shading in the human figure (Wallis & Bex, 2012).

Given the greater relevance of peripheral vision in athletic contexts, where monitoring off-centre stimuli is essential, this study focused specifically on peripheral processing. As a result, we did not include a central vision control condition, which however could have provided a useful baseline. Instead, we decided to investigate how our results might vary with eccentricity, although we did not find a significant effect. The lack of significant effects related to stimulus eccentricity could be due to the restricted range of eccentricities tested. Future studies could address this by using a wider display or immersive technologies, such as virtual reality environments (Toscani et al., 2019) to present stimuli at greater eccentricities and include central control condition to rule out the possibility that perceptual learning leads to a general improvement in perceptual ability across the entire visual field. Given the difficulties at recruiting different target populations in a between-participants design, we still think that our studies provided important evidence that outdoor athletes have better colour detection. This could be explained by their engagement with a rich, dynamic environment in which peripheral information plays an important role. Future studies could isolate the individual contributions – for example, low-level features such as local contrast and edges that may affect visual performance (Wallis & Bex, 2012), the extent of the visual field and higher-level aspects such as the complexity of outdoor environments in sport settings and dynamic interaction.

While our study does not establish a causal relationship between outdoor sport activities and enhanced perceptual performance, there is evidence for perceptual learning in adulthood: even brief periods of ocular deprivation can shift ocular dominance, highlighting the adaptability of the visual system (Lunghi et al., 2011).

Additionally, as the group of participants included athletes of varying competitive levels, ranging from recreational to more advanced, this variability in experience and training could have influenced performance outcomes. Amateur and professional athletes may differ in physiological and perceptual responses, suggesting that future studies should differentiate between these groups to better understand how expertise level impacts perceptual learning.

In conclusion, we found that outdoor athletes outperformed indoor athletes and non-athletes in peripheral detection tasks, providing evidence that exposure complex environments enhance visual perceptual skills. However, these enhancements were not specific to sport-related backgrounds or stimuli, suggesting that perceptual learning generalises across contexts. Our findings reinforce the idea that the adult brain is capable of perceptual adaptation and maintains plasticity, suggesting that perception can be altered outside of the critical formation period.
